# Composition of Micro-eukaryotes on the Skin of the Cascades Frog (*Rana cascadae*) and Patterns of Correlation between Skin Microbes and *Batrachochytrium dendrobatidis*

**DOI:** 10.3389/fmicb.2017.02350

**Published:** 2017-12-08

**Authors:** Jordan G. Kueneman, Sophie Weiss, Valerie J. McKenzie

**Affiliations:** ^1^Department of Ecology and Evolutionary Biology, University of Colorado Boulder, Boulder, CO, United States; ^2^Smithsonian Tropical Research Institute, Panama City, Panama; ^3^Department of Chemical and Biological Engineering, University of Colorado Boulder, Boulder, CO, United States

**Keywords:** microbiome, micro-eukaryote, network, skin, *Rana cascadae*, Batrachochytrium dendrobatidis, Cascades frog

## Abstract

Global amphibian decline linked to fungal pathogens has galvanized research on applied amphibian conservation. Skin-associated bacterial communities of amphibians have been shown to mediate fungal skin infections and the development of probiotic treatments with antifungal bacteria has become an emergent area of research. While exploring the role of protective bacteria has been a primary focus for amphibian conservation, we aim to expand and study the other microbes present in amphibian skin communities including fungi and other micro-eukaryotes. Here, we characterize skin-associated bacteria and micro-eukaryotic diversity found across life stages of Cascades frog (*Rana cascadae*) and their associated aquatic environments using culture independent 16S and 18S rRNA marker-gene sequencing. Individuals of various life stages of Cascades frogs were sampled from a population located in the Trinity Alps in Northern California during an epidemic of the chytrid fungus, *Batrachochytrium dendrobatidis*. We filtered the bacterial sequences against a published database of bacteria known to inhibit *B. dendrobatidis* in co-culture to estimate the proportion of the skin bacterial community that is likely to provide defense against *B. dendrobatidis*. Tadpoles had a significantly higher proportion of *B. dendrobatidis*-inhibitory bacterial sequence matches relative to subadult and adult Cascades frogs. We applied a network analysis to examine patterns of correlation between bacterial taxa and *B. dendrobatidis*, as well as micro-eukaryotic taxa and *B. dendrobatidis*. Combined with the published database of bacteria known to inhibit *B. dendrobatidis*, we used the network analysis to identify bacteria that negatively correlated with *B. dendrobatidis* and thus could be good probiotic candidates in the Cascades frog system.

## Introduction

Microbial symbionts of vertebrate hosts facilitate many biological processes, such as nutrient acquisition, host behavior, training of the adaptive immune system and protection against environmental pathogens (Bravo et al., 2011; Ezenwa et al., 2012; Tuddenham and Sears, 2015). Thus, microbial associations can contribute to host phenotypes and may influence survival (Fierer et al., 2012). The amphibian skin microbiome has become an active area of research due to the protective role that some bacteria provide against *Batrachochytrium dendrobatidis*, an emerging fungal skin pathogen (Harris et al., 2009; Kueneman et al., 2016). This aquatic pathogen, now the agent of a global pandemic, invades the keratinized epidermis of amphibians resulting in cell disruption and osmotic imbalances that can lead to death (Voyles et al., 2009). The use of 16S rRNA marker gene sequencing to characterize bacterial communities on amphibians has enabled many researchers to investigate the factors that shape those communities (McKenzie et al., 2012; Fitzpatrick and Allison, 2014; Jani and Briggs, 2014; Kueneman et al., 2014, 2015; Loudon et al., 2014; Walke et al., 2014). However, most studies have overlooked the fungal and micro-eukaryotic diversity that also occurs on amphibian skin and have yet to explore their function. Here, we use a parallel approach (18S rRNA marker gene sequencing) to explore micro-eukaryotic diversity alongside bacterial diversity on the skin of endangered Cascades frogs (*Rana cascadae*).

Bacterial-derived skin defenses have been experimentally shown to mitigate *B. dendrobatidis* infection (Harris et al., 2009; Jani and Briggs, 2014; Kueneman et al., 2016), however, interactions of other fungal taxa and micro-eukaryotes with *B. dendrobatidis* remain underexplored. Currently, it is unknown whether specific micro-eukaryotes may help mitigate infection of *B. dendrobatidis*. In plant systems, numerous experiments have found that some fungi are capable of excluding or reducing the effects of fungal pathogens. Examples include various leaf and root rots found in legumes, cacao, cotton, Dutch Elm, and fruit trees (Cotty and Bayman, 1993; Del Rio et al., 2002; Kabaluk et al., 2010). It is possible that symbiotic fungi on amphibians may be able to competitively exclude fungal pathogens through direct and indirect competition, however, this hypothesis remains to be tested on the amphibian skin.

In the absence of direct experimentation, network analyses provide one way to explore patterns of correlations within complex communities, identify microbes that negatively correlate with a pathogen, and infer possible antagonistic interactions (Rebollar et al., 2016a). While a negative correlation with a pathogen in a network does not confirm an antagonistic interaction, it is suggestive and should be tested independently. Microbes that are negatively correlated can help direct research to test specific interactions mechanistically and assess antibiosis that occurs with competing microbial taxa (Bletz et al., 2013). Previous field studies and lab experiments have demonstrated that individuals of species which have a higher proportion of their skin bacterial community composed of *B. dendrobatidis*-inhibitory taxa have lower abundance of *B. dendrobatidis* on their skin (Lam et al., 2010; Kueneman et al., 2016; Rebollar et al., 2016b). Thus, *B. dendrobatidis*-inhibitory bacteria negatively interacting with *B. dendrobatidis* within these networks are of special interest for conservation.

Cascades frogs are found in the Cascade and Olympic Mountains in the Pacific Northwest region of the United States. They have declined precipitously as a result of trout reintroduction and, more recently, *B. dendrobatidis* (Piovia-Scott et al., 2011). In Northern California, the Cascades frog population at Section Line Lake experienced a decline in juvenile frogs (>99%) attributed to *B. dendrobatidis* between 2009 and 2012 (Piovia-Scott et al., 2015). We had the opportunity to non-destructively sample the skin microbes from individuals in 2011, thus capturing a time point during an active *B. dendrobatidis* epidemic. Studying an epidemic in progress provides a unique perspective for examining the role of amphibian skin microbial communities in defense against pathogens, specifically by identifying microbes that have strong patterns of negative correlation with *B. dendrobatidis*. As Cascades frogs are imperiled, protective microbes can potentially offer new tools to augment the conservation of this species (Garcia et al., 2006; Piovia-Scott et al., 2015).

Here, we present a dataset that includes both the micro-eukaryotes and bacteria collected from the skin of wild *R. cascadae*. Our previous work contributed to a bacterial database of more than 1,200 isolates from amphibians around the globe that have demonstrated inhibitory action against *B. dendrobatidis* when tested in co-culture assays (Woodhams et al., 2015). No such database exists for the micro-eukaryotes, as of yet. We matched the bacterial sequence reads from the Cascades frogs against the *B. dendrobatidis-*inhibitory database to estimate the bacterial members of the skin community that are *B. dendrobatidis*-inhibitory. The aims of this study are threefold: (1) to characterize skin-associated micro-eukaryotes, including fungal skin communities among life-history stages of *R. cascadae*, (2) to examine correlational patterns among diverse microbes (bacteria and fungi), with a particular focus on interactions with the *B. dendrobatidis* pathogen, and (3) to identify candidate anti-*B. dendrobatidis* microbial taxa by leveraging the *B. dendrobatidis*-inhibitory sequence database.

## Materials and Methods

### Amphibian and Environmental Sampling

Individuals of *Rana cascadae* were caught and sampled in the Trinity Alps of Northern California, August of 2011. All individuals were captured using a dip net, handled with new nitrile gloves and sampled on the same day. In order to remove any transient microbes from the environment, each individual was rinsed twice with 50 mL of sterile water. Each amphibian was then sampled using a sterile cotton-tipped swab. Swabbing consisted of brushing over the entire ventral surface and limbs of the amphibian for 30 s. Tadpoles were swabbed uniformly over entire body for 30 s (McKenzie et al., 2012). All sampling was done non-destructively and individuals were released back into the lake. Lake water samples were collected by moving a swab through the water for 30 s at a depth of 40 cm. Sediment samples were collected via embedding the swab into the sediment for 30 s. Swabbing amphibians and the environment for the same amount of time (30 s) was part of the effort to standardize the sampling across sample types. Each swab was stored in its original sterile container and stored on ice for transfer to a 20°C freezer for storage until DNA extraction. Micro-eukaryotic communities from *R. cascadae* at Section Line, an alpine lake in the Trinity Alps of California, included tadpoles (*N* = 4), subadults (*N* = 6), adults (*N* = 11), sediment (*N* = 3), and lake water (*N* = 2). Permits and authorization were granted by California Fish and Game and the University of Colorado Institutional Animal Care and Use Committee.

### DNA Extraction and Sample Processing

DNA extraction was completed utilizing the MoBio Power Soil extraction kit (MoBio Laboratories, Carlsbad, CA, United States). We used the standard MoBio protocol with minor adjustments including incubating samples in 65°C for 10 min after the addition of C1, vortexing the PowerBead tubes horizontally for 2 min, and allowing solution C6 to sit on the filter for 5 min before the final elution (Fierer et al., 2008; Lauber et al., 2008). Extraction controls were included. The PCR recipe was comprised of: 12 μL PCR water, 10 μL 5 Prime Master Mix, 1.0 μL of the forward and reverse primers at 10 uM concentrations, 1.0 μL MgCl_2_, and 1.0 μL genomic DNA. For bacteria, PCR primers (515f/806r) were used to target the V4 region of the 16S rRNA gene and amplify region 533–786 in the *Escherichia coli* strain 83972 sequence (greengenes accession no. prokMSA_id:470367). The reverse PCR primer contained a 12-base error correcting Golay barcode developed in Caporaso et al. (2010). For micro-eukaryotes, primers 1391f/EukB were used (Amaral-Zettler et al., 2009). The PCR profiles included an initial denaturation step of 94°C for 3 min, followed by 35 cycles of 94°C for 45 s, 50°C for 60 s, and 72°C for 90 s, and final extension at 72°C for 10 min. The PCR was performed in triplicate and combined after amplification. Extraction controls were processed and showed no amplification. Amplicons were quantified using the Quant-IT Picogreen dsDNA reagent and were pooled into one sample per plate by combining equal concentrations of each amplicon. These pools of DNA were cleaned using the MoBio UltraClean PCR clean-up DNA purification kit. Following cleanup, samples were again quantified using PicoGreen reagent with equal concentrations and pooled together one final time before sequencing. A NanoDrop spectrophotometer was used to determine the purity and DNA concentration of this pool. Finally, prepared DNAs and control samples were sequenced using an Illumina HiSeq 2000 instrument at the BioFrontiers Institute Next-Generation Genomics Facility at the University of Colorado Boulder, Boulder, CO, United States.

### Sequence Filtering and Processing

**FIGURE 1:**
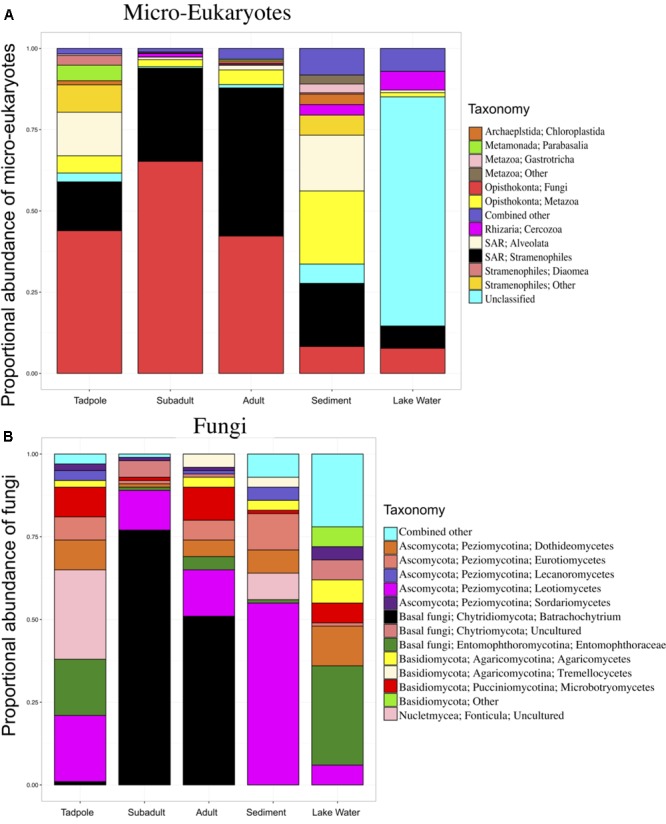
Skin micro-eukaryotic taxa on *Rana cascadae* from Section Line at each life stage, collected on the same day. The proportional abundance of **(A)** micro-eukaryote and **(B)** Fungal OTU sequences per major taxon across lifestages: tadpoles (*N* = 4), subadults (*N* = 6), adults (*N* = 11), sediment (*N* = 3), and lake water (*N* = 2). Data for both figures is based on the proportional abundance of each microbial taxon per individual. OTUs with lower than 0.5% total abundance were grouped into the category combined other. Average number of sequences per sample type; tadpoles (*N* = 213), subadults (*N* = 2,661), adults (*N* = 3,490), sediment (*N* = 125,818) and lake water (*N* = 58,062).

18S amplicons were sequenced on one Illumina MiSeq run at the University of Colorado Boulder, Boulder, CO, United States, yielding 150 base pair reads. All analyses were performed using QIIME v1.9.0 (Caporaso et al., 2010), unless otherwise stated. Sequences were filtered and assigned to samples using default settings and Cutadapt v1.2 (MARTIN, M) was used to trim primers from the combined forward and reverse sequences. Low abundance OTUs (i.e., less than 0.00005 proportional abundance) were removed (Bokulich et al., 2013) according to the subsampling open reference protocol (Rideout et al., 2014) using eukaryotic reference library Silva111 (Yilmaz et al., 2013). The no-template sequencing control was clear of unintended DNA amplification. Sequences were assigned to amphibian and environmental samples collected from the research location of Section Line Lake. Total micro-eukaryotic sequences were less abundant on tadpoles limiting the utility of rarefaction. Thus, to visualize the full extent of diversity within each lifestage, we use a proportional taxonomic abundance table (**Figures 1A,B**). However, we acknowledge that comparison across lifestages in **Figures 1A,B** is invalid, due to differences in library size. To give the viewer an idea of original library size used to make the proportions, average library size is indicated in the **Figure 1** legend. To confirm our approach, and compare micro-eukaryotic communities (18S) combined across all lifestages, we used a rarefaction depth of 330 micro-eukaryotic sequences per sample (Supplementary Table 1 and Supplementary Figures A,C). Rarefaction reduced samples sizes in the micro-eukaryotic dataset to tadpoles (*N* = 2), subadults (*N* = 6), adults (*N* = 9), sediment (*N* = 3), and lake water (*N* = 2). Rarefied data was used in part due to the >10x library size difference between tadpoles and other life stages (Weiss et al., 2017). Data was not rarefied in the correlation (Network) or differential abundance analyses (ANCOM) to maximize statistical power. Bacterial community (16S) sequence filtering and processing was done on a rarefied dataset of 19,900 sequences per sample. Bacterial samples tadpoles (*N* = 4), subadults (*N* = 8), adults (*N* = 12), sediment (*N* = 3), and lake water (*N* = 2) (**Figure 3**).

### Network Analysis

**FIGURE 2:**
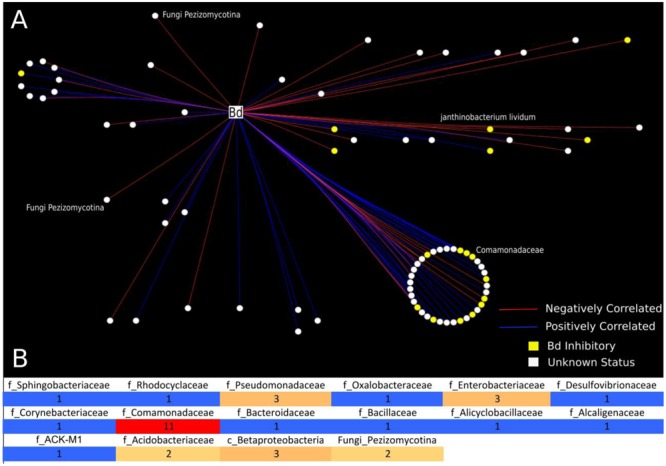
Analysis of bacterial and fungal OTUs that correlate with *B. dendrobatidis* found on Cascades frogs at Section Line. This analysis included 21 frog individuals for which we had successful sequencing yields for both 16S and 18S: tadpoles (*N* = 4), subadults (*N* = 6), adults (*N* = 11). **(A)** Network analysis is depicting only significantly correlating bacterial and fungal OTUs with *B. dendrobatidis.* All circular nodes represent OTUs (either bacteria or fungi) with significant correlation with pathogen *B. dendrobatidis* in the network. Red lines indicate negative correlation between two OTUs. Blue lines indicate positive correlation between two OTUs. Yellow nodes = (*B. dendrobatidis*-inhibitory), White nodes (Unknown Status). The large circle represents bacterial OTUs of the family Comomonadaceae; the smaller circle shows taxa clustering at the order level (Burkholderiales). Two fungal OTUs (Pezizomycotina) were found to negatively correlate with pathogen *B. dendrobatidis*. **(B)** Heatmap is depicting the number of negatively correlating OTUs between bacterial and fungal OTUs and fungal taxon *B. dendrobatidis*. All positively correlated interactions are excluded from the heatmap. The numbers in the heatmap indicate the frequency of negative correlations between bacterial and fungal groups calculated as the sum of negative OTU correlations per bacterial taxon and *B. dendrobatidis*, which are also depicted as red lines in **A**. Taxonomic levels are described as f = Family, c = Class, and Fungi = subdivision.

For the network analyses 16S and 18S datasets were merged and sediment and water samples were removed, resulting in 20 amphibian samples remaining with library sizes greater than 37,800. SparCC (Sparse Correlations for Compositional data) relies on sample proportions; therefore, analysis was done on the raw proportions without rarefying or other normalization. Then, OTUs not present in at least half the samples, and having total sum across samples less than 10, were removed prior to network analysis. This is because correlation detection performance degrades significantly with increased number of zero counts (Weiss et al., 2017). For SparCC in particular, performance degradation may be due to pseudo count addition. In this analysis ‘edges’ are positive and negative correlations with *B. dendrobatidis*. In the network, ‘edges’ with correlation values of 0.35 or higher were included. For SparCC (Friedman and Alm, 2012), correlation values are just as precise as *p*-value thresholds and computationally much faster (Weiss et al., 2017). In this study, we used default SparCC parameters. Since, we were mostly interested in *B. dendrobatidis*-inhibitory OTUs, only predicted ‘edges’ with *B. dendrobatidis* are shown in the network (**Figure 2**). A complete list of both positive and negative bacterial and micro-eukaryotic correlations with *B. dendrobatidis* can be found on the data repository DRYAD.

### *Batrachochytrium dendrobatidis*-Inhibitory Bacterial Isolates Database

Culturing of bacterial isolates from diverse amphibians across the globe, including *R. cascadae*, have been collated into a *B. dendrobatidis*-inhibitory database including ∼1,200 isolates that have been tested and shown to inhibit *B. dendrobatidis* in co-culture (Roth et al., 2013; Woodhams et al., 2015). The bacterial isolates within this database were Sanger sequenced (16S rRNA gene, 1,500 bp), then trimmed to 100 bp and used to pick OTUs with Greengenes reference database (August 2013 version). To explore the overlap between the *B. dendrobatidis*-inhibitory database and the naturally occurring microbial community of *R. cascadae*, we trimmed inhibitory OTUs based on sequences trimmed to the first 100 bp beyond primer 515f (GTGCCAGCMGCCGCGGTAA) to match the Illumina reads (819 isolates clustering to 304 OTUs). Then, we use expanded the dataset to include expected inhibitory OTUs within 0.1 Jukes-Cantor distances on the Greengenes phylogenetic tree (7,459 OTUs). This approach resulted in 7,459 unique expected inhibitory OTUs, forming the basis of the *B. dendrobatidis-*inhibitory database used in this study (available on Dryad). Using this strategy introduces some uncertainty, discussed below, however, it also provides a more comprehensive list of potential *B. dendrobatidis*-inhibitory taxa OTUs.

## Results

Total micro-eukaryotic sequences per sample ranged from 53 to 157,156, with a median observation level of 1,666. We observed 500 unique OTUs before rarefaction. Rarefication to a sequencing depth of 330 sequences per sample reduced the total number of unique OTUs to 255. Eukaryotic taxa found on amphibians included Fungi, Stramenopiles, and Metazoa. Analysis of Composition of Microbes (ANCOM) (Mandal et al., 2015) revealed six OTUs with significant differential abundance across life stages. Importantly, *B. dendrobatidis* was significantly lower on tadpoles and found highest on subadults. Both subadult and adult skin communities appeared to be dominated by pathogen *B. dendrobatidis* (**Figure 1B**). The five other taxa significantly differing across sample types were found to be more abundant in lake water (Supplementary Table 2). Qualitatively, fungi are the most abundant micro-eukaryotic taxa found on all life stages of *R. cascadae* and fungi shared between all amphibian life stages and sediment included Leotiomycetes and Eurotiomycetes. OTUs in the group Alveolata were found on both tadpoles and in the sediment (**Figure 1A**). Inter-sample variation for the proportional abundance of eukaryotic taxa across sample types was explored. Here, we see the proportional abundance of *B. dendrobatidis* is highest on all six subadults, compared with eight out of eleven adults revealing high proportional abundance of *B. dendrobatidis* (Supplementary Figure B). The combined patterns for proportion abundance using the full dataset are supported by the proportion abundance patterns using the rarefied dataset (Supplementary Figure C).

A list of the most abundant micro-eukaryotic OTUs found on *R. cascadae*, whether or not they were known to be pathogenic, and a brief description of where else they have been observed, is found in Supplementary Table 1. Surprisingly, we found nine OTUs of fungi, six OTUs of Stramenopiles, and five OTUs of Metazoa represented the majority of the micro-eukaryotic community. Of these 20 OTUs seven are believed to be pathogenic, and eight are believed to not have any negative consequences for the host. We did not find additional information for the remaining four OTUs.

The unequal and relatively small sample size of this dataset limits the strength of statistical measurements of diversity as well as identification of biologically relevant taxa. In particular, only a few tadpoles were found at this location and 18S sequencing yielded low library sizes for micro-eukaryotes. In contrast, 16S sequencing for bacteria yielded sufficient library sizes for comparison to other samples. The low library sizes for micro-eukaryotes warranted the multiple normalization methods described in the methods section. The normalization methods chosen were used to maximize information from the data while maintaining statistical integrity. We found Shannon diversity of micro-eukaryotes and fungi was higher in the sediment compared to amphibian samples. We also report no difference in Shannon diversity across the amphibian lifestages. These results are limited by the sample-sizes across groups and consequently the results and discussion of alpha diversity are otherwise excluded from the main text. A brief methods, results, and discussion of alpha diversity metrics are found in the Supplementary Figure A.

Using network analysis, we discovered that only two fungal OTUs, both Pezizomycota, were negatively correlated with *B. dendrobatidis* (**Figures 2A,B**). We observed that the majority of negative correlations occurred between bacterial OTUs that have not yet been challenged in co-culture (unknown status) and that the majority of both positively and negatively *B. dendrobatidis*-correlated bacterial taxa are in the class Burkholderiales and family Comomonadaceae (**Figure 2A**). Additional groups that negatively correlate with *B. dendrobatidis* are shown in a heat map (**Figure 2B**). Higher taxonomic resolution for both positive and negative correlation ‘edges’ can be found with supportive data materials on DRYAD. Specifically, taxa that negatively correlate with *B. dendrobatidis* and also match expected *B. dendrobatidis*-inhibitory database included Comamonadaceae (3) – OTU’s 270402, 536916, and 823696; Enterobacteriaceae (2) – OTUs 537871 and 783638; Pseudomonadaceae (1) – OTU 279948, and *Bacillus* (1) – OTU 321618. *Janthinobacterium lividum* (OTU 351280), a common antifungal bacterial isolate, was also found to negatively correlate with *B. dendrobatidis* (**Figure 2A**). Taxa listed here warrant increased attention for future conservation applications.

**FIGURE 3:**
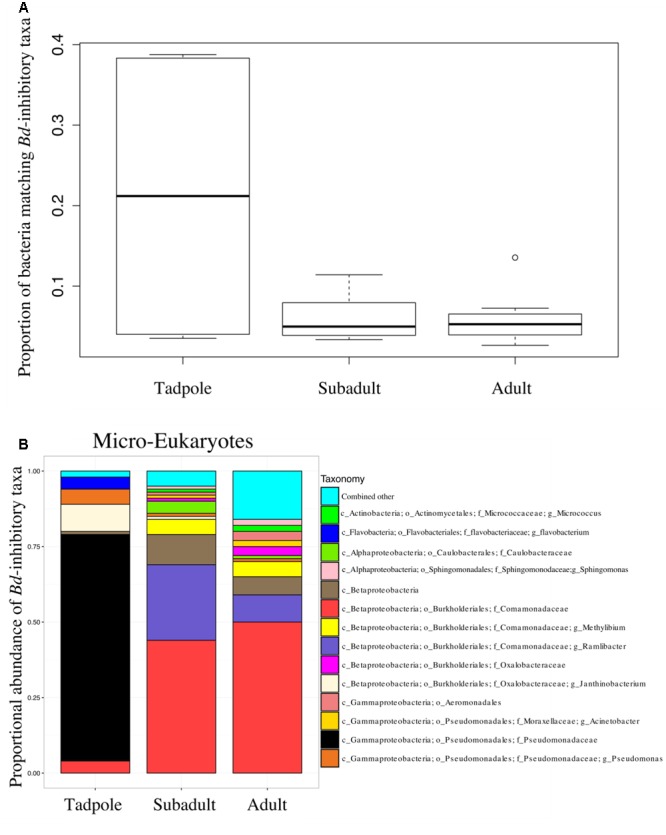
**(A)** The proportion of sequences that match the *B. dendrobatidis*-inhibitory database found on individuals. There is a higher proportion of the skin community that is *B. dendrobatidis*-inhibitory on tadpoles compared with subadults and adults; ANOVA, DF = 2, *F* = 6.143; *p* = 0.00793. Calculation for A and B are conducted on a rarefied dataset of 19,900 sequences per sample; Lifestages: tadpoles (*N* = 4), subadults (*N* = 6), adults (*N* = 11), sediment (*N* = 3), and lake water (*N* = 2). **(B)** *B. dendrobatidis*-inhibitory skin bacteria on *Rana cascadae* from Section Line at each life stage, collected on the same day. OTUs with lower than 1% total abundance are grouped into the category combined other.

The proportion of *B. dendrobatidis*-inhibitory bacteria was highest on tadpoles compared to subadults and adults (ANOVA, DF = 2, *F* = 6.14; *p* = 0.0079, **Figure 3**). The composition of expected *B. dendrobatidis*-inhibitory bacteria is shown in **Figure 3B**. Tadpole OTUs matching to *B. dendrobatidis*-inhibitory taxa were primarily in the family Pseudomonadaceae. Subadult OTUs primarily matched to members in the family Comamonadaceae, and more specifically an OTU in the genus *Ramlibacter*. Adult OTUs matched similarly to those of subadults, but also matched to low abundance inhibitory OTUs. Qualitatively, Enterobacteriaceae and *Bacillus* OTUs were comparatively very low in their sequence abundance, and Pseudomonadaceae, Comamonadaceae, and *Janthinobacterium lividum* were detected across all lifestages. A group significance (Kruskal-Wallis) test revealed that several OTUs matching to *Pseudomonas* as well as *J. lividum* were more abundant on tadpoles.

## Discussion

One previous study has also used 18S rRNA marker gene sequencing to examine the micro-eukaryotic skin community of amphibian boreal toad (*Anaxyrus boreas*) (Kueneman et al., 2015), thus allowing for some comparison. In *R. cascadae* we detect a higher proportion of *B. dendrobatidis*-inhibitory taxa on tadpoles compared with post-metamorphic individuals (**Figure 3**). This same pattern was found for *Anaxyrus boreas* (Kueneman et al., 2015). The dominant taxonomy of *B. dendrobatidis*-inhibitory bacteria found on tadpoles of both *R. cascadae* and *A. boreas* are not equivalent. This suggests that each species maintains its own unique protective assemblages (Pseudomonadales for *R. Cascadae* and Burkholderiales in *A. boreas*), which may have differential ability to inhibit diverse fungal groups. Comparing other micro-eukaryotes of *R. cascadae* to *A. boreas* qualitatively, we find that the class Stramenopiles is shared across life stages. Taxa in class Alveolata are present among tadpoles in the Cascades frog and the boreal toad, but are uniquely absent in post-metamorphic stages of Cascades frogs. Thus, we observe important similarities and differences in the diversity of micro-eukaryotes across amphibian life stages of two different amphibian families (Ranidae and Bufonidae).

Interactions between naturally occurring communities of microbial taxa on amphibian skin can be difficult to infer. However, we show that network analysis can be a useful tool to predict interactions between diverse taxa on the skin of *R. cascadae.* To date, the sequence database for *B. dendrobatidis*-inhibitory bacterial taxa (Woodhams et al., 2015) includes 37 bacterial isolates from *R. cascadae* (Roth et al., 2013), but only five isolates were found from the individuals sampled at Section Line in this dataset. Additionally, OTUs that match these five inhibitory isolates are relatively rare in our dataset of negatively correlating bacterial taxa with *B. dendrobatidis*. Thus, we utilize a *B. dendrobatidis*-inhibitory dataset, described in the methods section, which enables us to consider taxa that are closely related to *B. dendrobatidis-*inhibitory taxa isolated from other amphibian species. We report possible negative interactions that may occur between bacterial and micro-eukaryote taxa and *B. dendrobatidis* (**Figure 2**). Specific taxa, identified as negatively correlating with *B. dendrobatidis*, should be tested experimentally in the lab to confirm activity against *B. dendrobatidis*. After additional confirmation, these bacterial taxa can be referenced by the *B. dendrobatidis*-inhibitory database and better inform risk assessment of individuals, life stages, or populations of *R cascadae*, as well as other amphibian species. Only one bacterial taxon, *Janthinobacterium lividum*, identified here as negatively correlating with *B. dendrobatidis*, and matching the *B. dendrobatidis*-inhibitory database, has been tested as a probiotic on amphibians (Harris et al., 2009; Becker et al., 2011; Walke et al., 2015; Kueneman et al., 2016). However, its activity against *B. dendrobatidis* on various amphibian species offered mixed results, and its efficacy as a probiotic for *R. cascadae* remains to be explored *in vivo*. Intriguingly, *J. lividum* was found most abundantly on tadpoles. Additionally, isolates of the fungal group Pezizomycota (shown to negatively correlate with *B. dendrobatidis*) and more specifically Pezizomycetidae, should also be tested for their bioactivity against *B. dendrobatidis*. Taken together, taxa that negatively correlate with *B. dendrobatidis* and also match the *B. dendrobatidis*-inhibitory database may serve to direct additional studies aimed at conservation of *R. cascadae* and should be tested *in vivo.*

While the biology of many of the micro-eukaryotic taxa identified in this study remain unknown, several taxa identified, beyond *B. dendrobatidis*, have been previously shown to be antagonistic/pathogenic to free living organisms and could possibly influence the ecology of the frog skin microbiome (Supplementary Table 1). For example, *Entomophthora culicis* and *Leptolegnia caudata* are known parasites and pathogens of insects, respectively (Kerwin, 1982). Additionally, *Aphanomyces invadans* and *Karlodinium micrum* were found to be pathogens of fish and their eggs and *Saprolegnia* species are pathogens of both amphibians and fish, including their eggs (Bisht et al., 1996). Surprisingly, *Leptolegnia caudata* and *Aphanomyces invadans* were found to positively correlate with *B. dendrobatidis*. It is unknown at this point if the detection of these taxa signifies secondary infections or if they may have facilitated *B. dendrobatidis* infections. The ecology and consequence of the aforementioned taxa should be considered in future studies of *R. cascadae* health. Additionally, network analysis tools, while having reasonable false positive rates on OTUs with <50% zeroes, need improvement. Many tools have poor detection of ecological relationships other than mutualism and commensalism. SparCC, while attempting to account for compositions, is also one of the strongest network analysis techniques for detecting competitive mutual exclusion (Weiss et al., 2017). Detection of multispecies relationships is also difficult. In this study, increased library size would have provided more insight. Despite these weaknesses, correlation techniques have proven to be useful here and in a variety of additional studies.

In this study, we acknowledge the limitations to the predictive power of the *B. dendrobatidis*-inhibitory database. This database lacks sufficient information on bacterial isolates that do not exhibit bioactivity against *B. dendrobatidis*. This is because isolates cultured from amphibians were tested in co-culture with *B. dendrobatidis*, and then Sanger sequenced. Isolates that did not show inhibition against *B. dendrobatidis* are often not sequenced and thus not included in the database. Consequently, it is not possible to fully consider microbial interactions not captured by the database. To address the limitation that many *B. dendrobatidis* inhibitory microbes are also not present in the database we utilize an expanded list of OTUs to capture additional bacterial taxa that may be biologically important. Additional limitations to the predictive power of the *B. dendrobatidis-*inhibitory dataset exist: For example, (1) most isolates are cultured from adults, and thus the database may be biased toward microbial taxa that colonize the skin of post-metamorphic individuals (Woodhams et al., 2015). (2) In captivity, bacterial taxa may interact differently with *B. dendrobatidis* than they do in their natural environments. (3) Bacterial taxa that are inhibitory to *B. dendrobatidis* growth under certain temperature regimes are known to behave differently at other temperature regimes (Woodhams et al., 2014). (4) *B. dendrobatidis*-inhibitory taxa can act differently in the context of other microbes in the environment, or at certain densities (Woodhams et al., 2014). (5) Bacterial strains of the same species also behave differently with respect to *B. dendrobatidis*. (6) There is intrinsic bias for amplification of taxa based on the primers used. Specifically, pairs of primers for both bacteria and micro-eukaryotes have limitations for equal sequencing across all groups (Tedersoo et al., 2015; Tedersoo and Lindahl, 2016). (7) Lastly, many of the naturally occurring microbial OTUs are absent from the *B. dendrobatidis*-inhibitory database which limit the strength of its predictive power. The limitations listed here are universal to the state of extracting meaningful microbial interactions from microbial marker-gene surveys, when there is limited system specific knowledge. Indeed, while metagenomic tools may introduce error, they can also help to direct future studies to address specific knowledge gaps.

The role of skin microbial communities in protecting individuals from *B. dendrobatidis* is an area open to discovery that may help inform the defenses of *R. cascadae* against *B. dendrobatidis* and provide tools for enhanced conservation efforts of *R. cascadae* in the future. Important to the inferences made from this study, a particularly lethal strain of pathogen *B. dendrobatidis* has been isolated from Section Line Lake and tested in an experimental setting against *R. cascadae* reared from eggs. Individuals reared from eggs collected from Section Line survived longer and were healthier than other local populations tested against the Section Line *B. dendrobatidis* isolate (Piovia-Scott et al., 2015). In addition, individuals from Section Line survived longer against an extraneous *B. dendrobatidis* isolate, compared to other local populations tested (Piovia-Scott et al., 2015). The evidence shows that *R. cascadae* at Section Line interact with an aggressive strain of *B. dendrobatidis* and thus it is particularly important to understand their defenses against the pathogen. Unfortunately, subadults retained very high infection intensities (**Figure 1B**), and high mortality for this age group is observed in population demographics (Piovia-Scott et al., 2015). This dwindling age group is responsible for the continued decline of this remnant population of the Cascades frog. The function of skin microbial associations on amphibians has yet to be fully explored. This contribution begins to address knowledge gaps regarding the diversity and role of micro-eukaryotes in the health of amphibians, in identifying naturally occurring microbial interactions that may protect *R. cascadae* from fungal disease epidemics, and in direct conservation research for the most promising protective bacterial groups.

## Author Contributions

JK and VM conceived the study, JK collected the data, JK and SW analyzed the data, JK, VM, and SW wrote the paper.

## Conflict of Interest Statement

The authors declare that the research was conducted in the absence of any commercial or financial relationships that could be construed as a potential conflict of interest.
