# Supplementary material for: Composition of Micro-eukaryotes on the Skin of the Cascades Frog (Rana cascadae) and Patterns of Correlation between Skin Microbes and Batrachochytrium dendrobatidis
Source: Front Microbiol. 2017 Dec 8;8:2350. doi: 10.3389/fmicb.2017.02350 (PMC5727676; doi:10.3389/fmicb.2017.02350)
Supplement: Supplementary file 1 [file Data_Sheet_1.DOCX]

Supplemental Figure A

**Methods**

We used a rarefaction depth of 330 micro-eukaryotic sequences per sample. Rarefaction reduced samples sizes in the micro-eukaryotic dataset to tadpoles (N=2), subadults (N=6), adults (N=9), sediment (N=3), and lake water (N=2). Rarefied data was used in part due to the > 10x library size difference between tadpoles and other life stages (Weiss et al. 2017).

**Results**

Analyses of Shannon diversity for “Micro-Eukaryote Shannon Diversity” and “Fungal Shannon Diversity” revealed highest diversity in the sediment (ANOVA, F=12.39; p =< 0.0001) and (ANOVA, F=10.33; p =< 0.0001) respectively. No significant differences were found across lifestages for “Micro-Eukaryote Shannon Diversity” or for “Fungal Shannon Diversity”.

Supplemental Figure 1


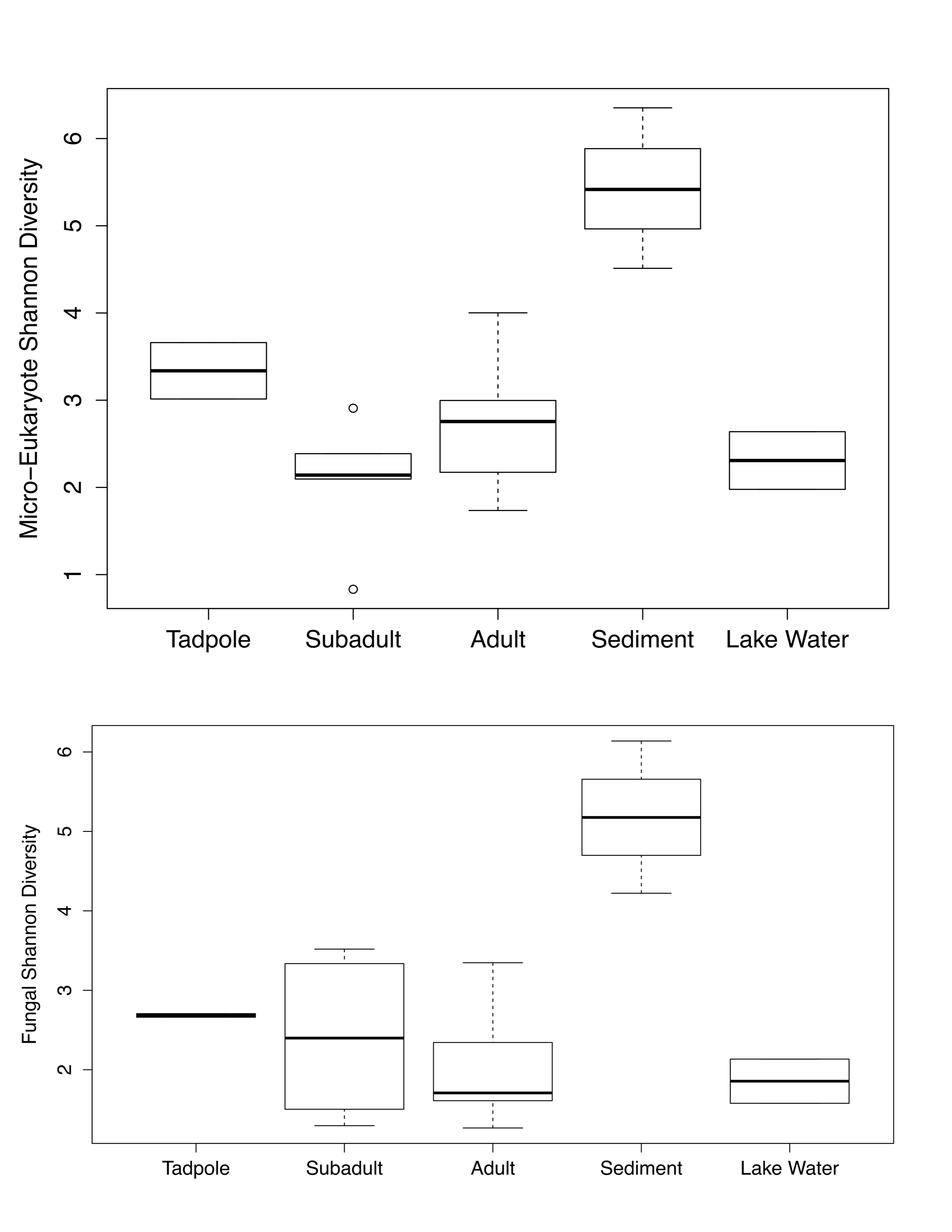


Alpha diversity (Shannon) of “Micro-Eukaryote Shannon Diversity” and “Fungal Shannon Diversity”, analyzed across life stages and environmental samples. Highest diversity was found in the sediment “Micro-Eukaryote Shannon Diversity” ANOVA, F=12.39; p =< 0.0001 and “Fungal Shannon Diversity” ANOVA, F=10.33; p =< 0.0001. No difference was found between lifestages.

**Discussion**

The unequal and relatively small sample size of this dataset, as well as small library sizes for tadpoles limits the strength of statistical measurements of diversity. Considering these limitations, we report in the main text that alpha diversity comparisons suggest that the highest diversity is in sediment and no difference was found across lifestages.
