# Supplementary material for: Composition of Micro-eukaryotes on the Skin of the Cascades Frog (Rana cascadae) and Patterns of Correlation between Skin Microbes and Batrachochytrium dendrobatidis
Source: Front Microbiol. 2017 Dec 8;8:2350. doi: 10.3389/fmicb.2017.02350 (PMC5727676; doi:10.3389/fmicb.2017.02350)
Supplement: Supplementary file 3 [file Data_Sheet_3.DOCX]

Supplemental Figure C


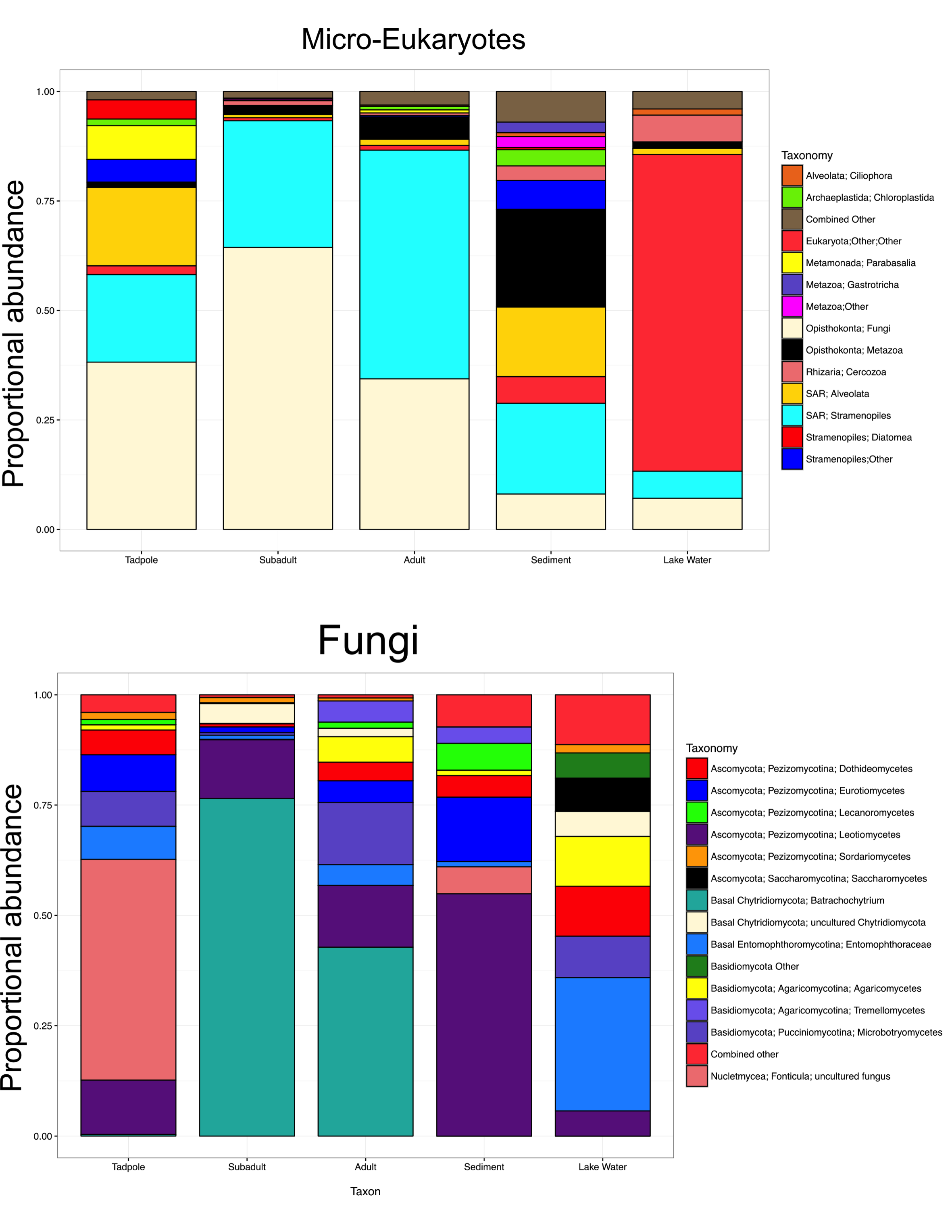


Skin micro-eukaryotic taxa on *Rana cascadae* from Section Line at each life stage, collected on the same day. The proportional abundance of (A) micro-eukaryote and (B) fungal OTU sequences per major taxon across lifestages: tadpoles (N=2), subadults (N=6), adults (N=9), sediment (N=3) and lakewater (N=2). Data for both figures is based on the proportional abundance of each microbial taxon per individual. OTUs with lower than 0.5% total abundance were grouped into the category Combined other.
