# Supplementary material for: Composition of Micro-eukaryotes on the Skin of the Cascades Frog (Rana cascadae) and Patterns of Correlation between Skin Microbes and Batrachochytrium dendrobatidis
Source: Front Microbiol. 2017 Dec 8;8:2350. doi: 10.3389/fmicb.2017.02350 (PMC5727676; doi:10.3389/fmicb.2017.02350)
Supplement: Supplementary file 5 [file Data_Sheet_5.DOCX]

**References: Supplemental Table 1**

Bary, A. (1888). Species der Saprolegnieen. Botanische Zeitung. 46:597-653

Barr, D.J., (1990). Phylum chytridiomycota. *Handbook of Protoctista*. Jones & Bartlett, Boston. 454-66.

Bisht, G.S., Joshi. C.H., Khulbe. R.D., (1996). Watermolds: Potential biological control agents of malaria vector Anopheles culicifacies. *CURRENT SCIENCE-BANGALORE*-. 70:393-5.

Blazer, V.S., Lilley, J.H., Schill, W.B., Kiryu, Y., Densmore. C.L., Panyawachira, V., and Chinabut, S. (2002). Aphanomyces invadans in Atlantic menhaden along the east coast of the United States. *Journal of Aquatic Animal Health*. 14(1):1-0.

Boekhout, T., Roeijmans, H., and Spaay, F. (1995). A new pleomorphic ascomycete, Calyptrozyma arxii gen. et sp. nov., isolated from the human lower oesophagus. *Mycological research*. (10):1239-46.

Buttrey, B.W. (1954). Morphological variations in Tritrichomonas augusta (Alexeieff) from amphibia. *Journal of Morphology*, *94*(1), pp.125-163.

*Capronia pilosella* (Karst.) E. Müller, Trans. Br. Mycol. Soc. 88: 63. 1987.Basionym: *Sphaeria pilosella* (Karst.), Mycol. Fenn. 2: 96. 1873. http://www.bcrc.firdi.org.tw/fungi/fungal_detail.jsp?id=FU200802010025

Deeds, J.R., Terlizzi, D.E., Adolf, J.E., Stoecker, D.K., and Place, A.R. (2002). Toxic activity from cultures of Karlodinium micrum (Gyrodinium galatheanum)(Dinophyceae)—a dinoflagellate associated with fish mortalities in an estuarine aquaculture facility. *Harmful Algae*.1(2):169-89.

Dennis, P.R. (2009). Dragonflies and Damselflies of the West*. Princeton University Press. ISBN 0-691-12281-4.*

Donk, M.A*.* (1966). *Check list of European hymenomycetous heterobasidia. Persoonia. 4: 145–335.*

Deasey, M.C., and Olive, L.S. (1981). Role of Golgi apparatus in sorogenesis by the cellular slime mold Fonticula alba. *Science.* 213(4507):561-3.

Fell, J.W., Kurtzman,. CP., Tallman, A.S., and Buck JD. (1998). Rhodosporidium fluviale sp. nov., a homokaryotic red yeast from a subtropical brackish environment. *Mycologia*. 1:560-4.

Hogan, C.M. (2008) Rough-skinned Newt (Taricha granulosa), Globaltwitcher.

Kerwin, J.L. (1982). Chemical control of the germination of asexual spores of Entomophthora culicis, a fungus parasitic on dipterans. *Microbiology*. 128(9):2179-86.

Lumbriculus variegatus. (2017, April 17). In *Wikipedia, The Free Encyclopedia*. Retrieved 00:49, June 26, 2017

Mann, D.G., McDonald, S.M., Bayer, M.M., Droop, S.J., Chepurnov, V.A., Loke, R.E., et al. (2004). The Sellaphora pupula species complex (Bacillariophyceae): morphometric analysis, ultrastructure and mating data provide evidence for five new species. *Phycologia*. 43(4):459-82.

Mayer, K. (2000) Saprolegnia: There’s a fungus among us. *OSU Department of Fisheries and Wildlife*.

Ruthig, G.R., (2009). Water molds of the genera Saprolegnia and Leptolegnia are pathogenic to the North American frogs Rana catesbeiana and Pseudacris crucifer, respectively. *Diseases of aquatic organisms*, *84*(3), pp.173-178.

Ramamurthi, C.S., Korf. R.P., and Batra. L.R. (1957). A revision of the North American species of Chlorociboria (Sclerotiniaceae). *Mycologia*. 49(6):854-63.

Skerratt, L.F., Berger, L., Speare, R., Cashins, S., McDonald, K.R., Phillott, A.D., et al. (2007). Spread of chytridiomycosis has caused the rapid global decline and extinction of frogs. *EcoHealth*, 4(2), pp.125-134.

Wilke, U. (1954). Mediterrane Gastrotrichen. *Zool. Jahrb*., Abt. Syst.,
82: 497-550.
