# Supplementary material for: Composition of Micro-eukaryotes on the Skin of the Cascades Frog (Rana cascadae) and Patterns of Correlation between Skin Microbes and Batrachochytrium dendrobatidis
Source: Front Microbiol. 2017 Dec 8;8:2350. doi: 10.3389/fmicb.2017.02350 (PMC5727676; doi:10.3389/fmicb.2017.02350)
Supplement: Supplementary file 6 [file Data_Sheet_6.docx]

Supplementary Table 2

Shows OTUs detected as differentially abundant across lifestages and the environmental using Analysis of Composition of Microbes (ANCOM).
